# KDF1 Promoted Proliferation, Migration and Invasion of Lung Adenocarcinoma Cells through Activating STAT3 and AKT Pathway

**DOI:** 10.3390/biomedicines11123194

**Published:** 2023-12-01

**Authors:** Yi-Qing Guo, Mei-Fu Gan, Jia-Qian Bao, Han-Xi Zhou, Jing Yang, Chuan-Jing Dai, Jing-Min Zheng

**Affiliations:** Department of Pathology, Taizhou Hospital, Wenzhou Medical University, Linhai 317000, China

**Keywords:** AKT, KDF1, lung adenocarcinoma, STAT3

## Abstract

KDF1 has been reported to be correlated with carcinogenesis. However, its role and mechanism are far from clear. To explore the possible role and underlying mechanism of KDF1 in lung adenocarcinoma (LUAD), we investigated KDF1 expression in LUAD tissues and the influence of KDF1 in the phenotype of LUAD cells (A549 and PC-9) as well as the underlying mechanism. Compared to non-tumor lung epithelial cells, KDF1 was upregulated in the cancer cells of the majority of LUAD patients, and its expression was correlated with tumor size. Patients with enhanced KDF1 in cancer cells (compared with paired adjacent non-neoplastic lung epithelial cells) had shorter overall survival than patients with no increased KDF1 in cancer cells. Knockdown of KDF1 inhibited the migration, proliferation and invasion of LUAD cells in vitro. And overexpression of KDF1 increased the growth of the subcutaneous tumors in mice. In terms of molecular mechanisms, overexpression of KDF1 induced the expression of AKT, p-AKT and p-STAT3. In KDF1-overexpressing A549 cells, inhibition of the STAT3 pathway decreased the level of AKT and p-AKT, whereas inhibition of the AKT pathway had no effect on the activation of STAT3. Inhibition of STAT3 or AKT pathways reversed the promoting effects of KDF1 overexpression on the LUAD cell phenotype and STAT3 inhibition appeared to have a better effect. Finally, in the cancer cells of LUAD tumor samples, the KDF1 level was observed to correlate positively with the level of p-STAT3. All these findings suggest that KDF1, which activates STAT3 and the downstream AKT pathway in LUAD, acts as a tumor-promoting factor and may represent a therapeutic target.

## 1. Introduction

Lung cancer causes high human morbidity and death each year [1]. As a major subtype of lung cancer, lung adenocarcinoma (LUAD) comprises approximately 40% of newly diagnosed lung cancer cases [2,3]. Despite the huge progress that has been made in the past decades in the recognition and treatment of LUAD, there is still a long way to go before we fully comprehend the precise molecular mechanisms underlying the onset and progression of the disease. Most patients with LUAD are asymptomatic at an early stage, making early detection of the disease difficult. Thus, many patients are already at an advanced stage when first diagnosed. The prognosis of the disease is far from ideal, with the five-year overall survival (OS) rate being about 15% [4]. There is still an urgent need to further identify LUAD-associated molecules and investigate their pathological significance in the disease in order to promote our understanding of molecular mechanisms and search for novel prognostic molecular markers and therapeutic targets.

Keratinocyte differentiation factor 1 (KDF1) is a protein initially found to have an important role in regulating epidermal development. KDF1 mutant mice exhibit abnormal basal progenitor cell proliferation and differentiation, resulting in defects in epidermal structure and function [5]. The function of KDF1 revealed in regulation of cell differentiation and proliferation suggests that it may also have roles in the pathogenesis of cancers, a group of diseases that occur due to uncontrolled proliferation. In a previous study, we observed that KDF1 was downregulated in clear cell renal cell carcinoma and might act as a suppressing factor in the disease [6]. In contrast, KDF1 was reported to play an oncogenic role in epithelial ovarian cancer recently [7]. Based on these studies, KDF1 may not play a single regulatory role in the carcinogenesis process. Whether it has a tumor-promoting or tumor-suppressing function depends on the specific type of tumor cells. And from the literature we reviewed, no specific role of KDF1 in LUAD has been reported. Here, we explored the expression characteristics and functions of KDF1 in LUAD, as well as the possible molecular mechanisms.

## 2. Materials and Methods

### 2.1. Patients

A total of 160 patients, comprising 56 males and 104 females, were enrolled. The patients were aged between 28 and 81 years (average 62.1 ± 10.3 years. All the patients were hospitalized at the Department of Cardiothoracic Surgery, Taizhou Hospital from January 2013 to June 2015. All the patients were confirmed for LUAD by histological examination after partial pneumonectomy. Exclusion criteria included patients with a history of other malignancies or preoperative antitumor therapy. Additionally, patients with other histological subtypes (mixed subtypes) were not included. The 8th edition AJCC/UICC stage classification for lung cancer was followed in determining the pathological stage of each patient. The follow-up records and medical records of each patient were carefully reviewed and the clinicopathologic data were collected. Informed consent has been obtained from each patient. All the ethical standards on human experimentation were well followed.

### 2.2. Immunohistochemistry

Immunohistochemistry was carried out in the usual manner [6]. Antibodies used in this study are KDF1 antibody (Cat.No. PA5-55926, Thermo Scientific, Carlsbad, CA, USA, dilution 1:200), AKT antibody (Cat.No. 4685S, CST, Danvers, MA, USA, dilution 1:200), phosphorylated AKT (p-AKT) antibody (Cat.No. 4060S, CST, Danvers, MA, USA, dilution 1:200), phosphorylated STAT3 (p-STAT3) antibody (Cat.No. 9145S, CST, Danvers, MA, USA, dilution 1:200), Ki-67 antibody (Cat.No. 790-4286, Roche, AZ, USA, dilution 1:2) and horseradish-peroxidase-labeled second antibody (Cat.No. RQ7025, Quanhui, Guangdong, China) in a blind manner. Two experienced researchers scored the KDF1 level according to the staining intensity and percentage of positive cells: 1, weak; 2, medium; 3, strong. The following criteria were applied in scoring the level of KDF1 in tumor tissue sections: 1: weak staining or medium staining in less than 50% of tumor cells or strong staining in less than 25% of cancer cells; 2: medium staining of in 50–75% of cells or strong staining in 25–50% of tumor cells; 3: medium staining in more than 75% of cells or strong staining in more than 50% of cells.

To evaluate the proportion of Ki-67-positive cells, in each section, 15 non-overlapping high-power field images were randomly selected. The total nuclei number and Ki-67-positive nuclei number were carefully counted. Then, the Ki-67-positive nuclei number was divided by the total nuclei number and the proportion of Ki-67-positive cells was obtained.

### 2.3. Cell Culture and Treatment

All cell lines were from the Cell Bank of the Chinese Academy of Science (Shanghai, China). To obtain cells overexpressing KDF1, the cells were infected with Lenti-KDF1, a lentivirus constructed by cloning the KDF1 coding sequence into multiple pCDH-CMV-MCS-EF1 cloning sites. As for cells with low expression of KDF1, the cells were infected with Lenti-shKDF1, a recombinant lentivirus that was made by cloning the KDF1 shRNA (5′-GAGGAGTACTATTCTTTCCATCTCGAGATGGAAAGAATAGTAC-TCCTCTTTTTT-3′) into multiple pLKO-1-mCherry-P2A-Neo cloning sites. Two control lentiviruses, Lenti-O-VC, which was produced by using a pCDH-CMV-MCS-EF1 empty vector, and Lenti-sh-VC, which was made by cloning a scrambled shRNA into the multiple cloning sites of pLKO-1-mCherry-P2A-Neo, were also used in the experiments.

We performed transfection when the cells grew to about 60% confluence using a multiplicity of infection (MOI) of about 3.0. Puromycin (5 μg/mL) or G418 (2 mg/mL) were used to screen the stably transfected cells.

We used LY294002 (Cat.No HY-10108, MedChemExpress LLC, Monmouth Junction, NJ, USA) and Stattic (Cat.No HY-13818, MedChemExpress LLC, Monmouth Junction, NJ, USA) to inhibit the activation of the AKT and STAT3 pathway, respectively.

### 2.4. Quantitative Real-Time Polymerase Chain Reaction (qPCR)

Total RNA was purified using TRIzol^®^ reagent (Cat.No. 9109, Thermo Scientific, Waltham, MA, USA). cDNA was synthesized by using a PrimeScript™ kit (Cat.No. RR01AM, Takara Biotechnology, Dalian, China). Primers used for amplifying KDF1 fragment were: KDF1-sense, 5′-GTACCCAGCAAGCCATGA-3′ and KDF1-antisense, 5′-CTCCCAGAAAGGGTGTGG-3′. Primers used for amplifying 18S RNA fragment were: 18S-sense, 5′-TTTCTCGATTCCGTGGGTGG-3′ and 18S-antisense, 5′-AGCATGCCAGAGTCT-CGTTC-3′. Primers used for amplifying MMP1 fragment were: MMP1-sense, 5′-ATGTGGAGTGCCTGATGTGG-3′ and MMP1-antisense, 5′-TTGTCCCGATGATCTCCCCT-3′. Primers used for amplifying MMP2 fragment were: MMP2-sense, 5′-TACAGGATCATTGGCTACACACC-3′ and MMP2-antisense, 5′-GGTCACATCGCTCCAGACT-3′. Primers used for amplifying MMP7 fragment were: MMP7-sense, 5′-GGAACAGGCTCAGGACTATCT-3′ and MMP7-antisense, 5′-TTTCCTGAAATGCAGGGGGA-3′. Primers used for amplifying MMP9 fragment were: MMP9-sense, 5′-TGTACCGCTATGGTTACACTCG-3′ and MMP9-antisense, 5′-GGCAGGGACAGTTGCTTCT-3′. Primers used for amplifying MMP15 fragment were: MMP15-sense, 5′-TTCGGGGTACGAGTGAAAGC-3′ and MMP15-antisense, 5′-ATGGTGGTTGTTCCACTTCCT-3′. The 2^−ΔΔCt^ method was used in calculating KDF1 mRNA levels.

### 2.5. Western Blot Analysis

Western blot was performed as usual [6]. Antibodies used included: KDF1 antibody (1:1000), ERK antibody (Cat.No. 4695S, CST, Danvers, MA, USA, 1:1000), phosphorylated ERK (p-ERK) antibody (Cat.No. 4370S, CST, MA, USA, 1:1000), p38 antibody (Cat.No. 8690S, CST, Danvers, MA, USA, 1:1000), phosphorylated p38 (p-p38) antibody (Cat.No. 4511S, CST, MA, USA, 1:1000), AKT antibody (1:1000), p-AKT antibody (1:1000), Iκκα antibody (Cat.No. 11930S, CST, Danvers, MA, USA, 1:1000), STAT3 antibody (Cat.No. 9139S, CST, MA, USA, 1:1000), p-STAT3 antibody (1:1000), HRP-labeled goat antirabbit (Cat.No. ARG65351, ARIGO, Taiwan, China, 1:10,000) and goat antimouse (Cat.No. ARG65350, ARIGO, Taiwan, China, 1:10,000).

### 2.6. Cell Vitality Assay

Cell vitality analysis was carried out by using a CCK-8 kit (Cat.No. C0039, Beyotime Institute of Biotechnology, Shanghai, China) using the method recommended by the producer.

### 2.7. Colony-Forming Assay

Cells were seeded into 35 mm dishes (1000 cells/dish) and incubated for 8 days at 5% CO_2_ 37 °C. After being fixed with 4% formaldehyde and washed with PBS, the cell clones were stained by using 4% crystal violet. The number of visible cell clones was carefully counted. To evaluate clone size, under a microscope, 50 clones were randomly photographed in a dish, the cell number in each clone was carefully counted and the average number of cells per clone in each dish was calculated and used as the size of the clone in the dish.

### 2.8. EdU Incorporation Test

Using the method recommended by the producer, EdU incorporation tests were carried out using a BeyoClick™ EdU Cell Proliferation Kit with Alexa Fluor 594 (Cat.No. C0078L, Beyotime Institute of Biotechnology, Shanghai, China).

### 2.9. Wound-Healing Assay

Wounds on the monolayer of cells were produced using a sterile micropipette tip. After removing the detached cells by washing with serum-free medium, the cells were maintained in RPMI 1640 medium containing 0.5% FBS (the cells did not proliferate under this condition). Pictures of scratches were taken at regular time points, the unhealed areas of the scratches were measured by using ImageJ software (version no. 1.8, NIH, Bethesda, MD, USA) and finally the healing areas were calculated and compared.

### 2.10. Matrigel Invasion Assay

To assess the invasion ability of the cells, a Matrigel precoated Transwell system (8 μm pores) (BD Biosciences, San Jose, CA, USA) was employed. Briefly, 20,000 cells re-suspended in serum-free medium (200 μL) were seeded in the upper chamber and a total of 600 μL cell culture medium containing 10% FBS was added to the lower chamber. After being cultured for 48 h, the cells on the membrane were fixed and stained with crystal violet. Pictures were taken with a microscope and the number of invaded cells was counted.

### 2.11. Tumorigenicity Assay

Eighteen BALB/C nude mice (male) were randomly divided into three groups: A549 cell group (A group), control lentivirus-transfected A549 cell group (ANC group) and KDF1-overexpressing A549 cell group (AK). For each mouse, 1 × 10^6^ cells (A549 cells for A group; A549 cells infected with the control virus (Lenti-O-VC) for ANC group and A549 cells with KDF1 overexpressed for the AK group) suspended in 100 μL RPMI 1640 medium containing 50% Matrigel were subcutaneously injected into the side of the armpit. After 6 weeks, we euthanized the mice and isolated and weighed the tumor. 

### 2.12. Statistical Analysis

SPSS software (version no. 19.0, IBM, Armonk, NY, USA) was used in data analysis except as noted. Comparisons between multiple groups were carried out by using one-way ANOVA. Comparison of KDF1 protein levels in the cancer cells of LUAD tumor tissue and adjacent non-tumor lung epithelial cells (represented by a score) was carried out using Mann–Whitney U test. A *t*-test was used in other comparisons between two groups. Spearman correlation was performed in the correlation analysis of KDF1 level with the level of p-STAT3 in LUAD tissues. Survival analysis was carried out by using the Kaplan–Meier method and log-rank test.

## 3. Results

### 3.1. KDF1 Was Increasingly Expressed in the Tumor Tissues of LUAD Patients

**Figure 1:**
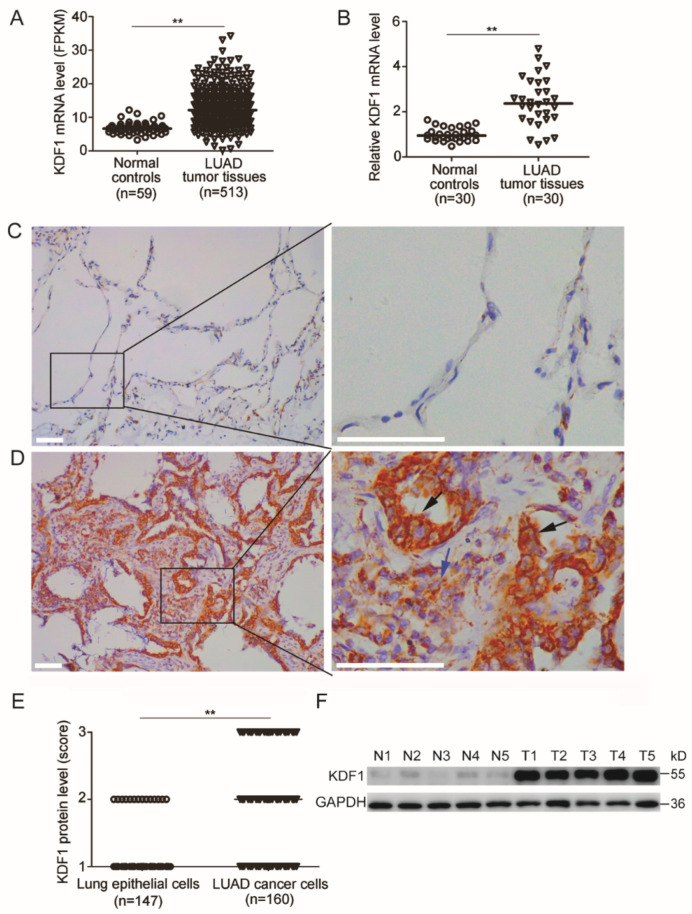
Expression of KDF1 in lung adenocarcinoma (LUAD) tissue. The expression of KDF1 was analyzed by using the data downloaded from TCGA database, quantitative PCR (qPCR), immunohistochemistry and Western blot methods. (**A**) Results of analysis based on the transcription data downloaded from TCGA database. (**B**) Results of qPCR analysis. (**C**) A representative image of KDF1 immunohistochemical staining in adjacent non-tumor lung tissues shows that KDF1 was weakly expressed by lung epithelial cells. (**D**) A representative image of KDF1 immunohistochemical staining in LUAD tumor tissues showing that enhanced KDF1 was expressed in the cancer cells (shown by black arrow) and some non-cancer cells (shown by blue arrow). (**E**) Results of statistical analysis of KDF1 protein level in non-tumor epithelial cells and LUAD cancer cells based on immunostaining intensity score. (**F**) Results of Western blot showing a marked increase in the expression level of KDF1 protein in LUAD tumor tissue samples in comparison with the paired non-tumor lung tissue samples. N1–N5: Non-tumor lung tissue samples; T1–T5: LUAD tissue samples. ** *p* < 0.01.

We first examined the level of KDF1 mRNA in LUAD tumor tissues using the data downloaded from TCGA online database. We observed significantly increased KDF1 mRNA in the tumor tissues of LUAD patients compared with that in the controls (Figure 1A). The increased KDF1 mRNA in LUAD tumor tissues was also proved by the results of our quantitative RT-PCR analysis, in which the RNA from 30 tumor tissue samples and paired non-tumor tissue samples was used (Figure 1B).

As an upregulated KDF1 mRNA level does not necessarily mean an upregulated KDF1 protein level, to determine whether KDF1 protein was also increasingly expressed in LUAD tumor tissue, immunohistochemistry was further performed. Of the 160 LUAD cases analyzed, 147 had both tumor tissue and paired non-tumor tissue specimens, and the other 13 had only tumor tissue specimens. We observed that in most of the non-tumor tissues, immunostaining for KDF1 was very weak (Figure 1C). In contrast, immunostaining for KDF1 was much higher in most tumor tissues (Figure 1D). The enhanced KDF1 in the tumor tissues was observed in the cancer cells (Figure 1D, shown by a black arrow) as well as in some non-cancer cells (Figure 1D, shown by a blue arrow). KDF1 protein was distributed mainly in the cytoplasm.

According to the KDF1 immunostaining intensity in the cancer cells, 32, 67 and 61 patients were respectively scored as 1, 2 and 3. In contrast, the expression levels of KDF1 in non-tumor lung epithelial cells of 133, 14 and 0 patients were scored 1, 2 and 3, respectively (Figure 1E). Among the 147 cases that have both tumor tissue and adjacent non-tumor lung tissue, 108 cases were found to have obviously higher immunostaining for KDF1 in the cancer cells than in the paired adjacent non-tumor epithelial cells. In the other 39 cases, the immunostaining intensity of KDF1 was similar to that of the adjacent pulmonary epithelial cells.

To confirm the results of immunohistochemistry, we further detected the KDF1 protein level in five LUAD tumor tissue samples in comparison with the paired adjacent non-tumor lung tissues. We observed a markedly increased KDF1 protein level in the tumor tissue samples compared to the non-tumor tissue samples (Figure 1F), thus proving the results of our immunohistochemistry.

### 3.2. Expression of KDF1 in Other Cancers

**Figure 2:**
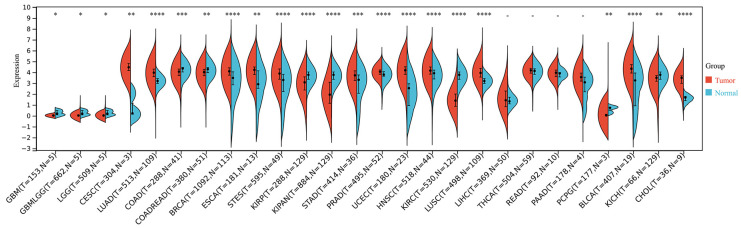
Results of pan-cancer analysis of KDF1. The pan-cancer analysis was performed by using the SangerBox platform. * *p* < 0.05; ** *p* < 0.01; *** *p* < 0.001; **** *p* < 0.0001.

To obtain a full view of KDF1 expression in cancers, we carried out a pan-cancer analysis using the SangerBox platform (http://sangerbox.com/ (accessed on 26 June 2023)). As demonstrated in Figure 2, KDF1 was observed to be differentially expressed in 22 out of the 26 types of cancers analyzed. It was upregulated in 12 cancers and downregulated in 10 cancers, indicating that KDF1 might be extensively involved in the pathogenesis and carcinogenesis.

### 3.3. Association between the Level of KDF1 and Clinicopathological Parameters

Patients were divided into different pairs of groups based on age, sex, T classification, disease stage tumor size and the presence of metastasis. Then, these groups were further divided into three groups according to the immunostaining score, thus revealing the relationship between KDF1 expression and clinicopathological parameters. As shown in Table 1, cancer cells of the large tumor group were found to harbor a higher KDF1 expression level compared to the smaller tumor group. Of note, although not reaching the statistical significance level (*p* = 0.065), the cancer cells in the advanced-stage group tended to have increased KDF1 compared to the early-stage group (Table 1). No significant difference in the cancer cells’ KDF1 protein level was observed between the groups of patients with different age, gender, T classification and metastasis (Table 1).

### 3.4. Patients with Increased KDF1 in the Cancer Cells Had Shorter OS

**Figure 3:**
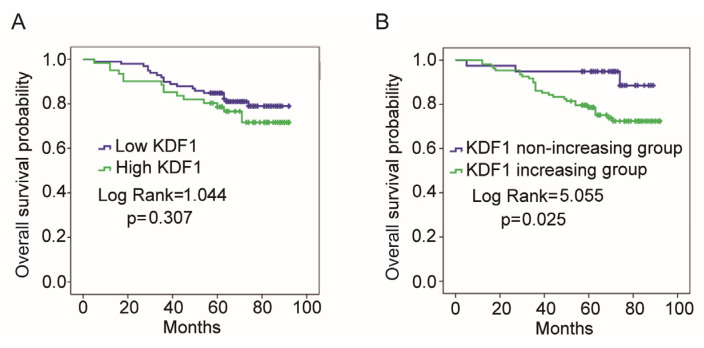
Results of overall survival (OS) analysis. According to the immunostaining intensity of KDF1 in cancer cells and whether cancer cells have higher KDF1 level compared with the paired adjacent non-neoplastic lung epithelial cells, we divided the patients into the following two pairs of groups: low- and high-KDF1 groups; KDF1-increasing and KDF1-non-increasing groups. Then, we compared the OS between each pair of groups by using the Kaplan–Meier method. (**A**) Results of comparison of the OS between the low-KDF1 group and the high-KDF1 group. (**B**) Results of comparison of the OS between the KDF1-increasing and KDF1-non-increasing groups.

According to the absolute immunostaining intensity of KDF1, patients were divided into low- (with KDF1 score being 1 or 2) and high-KDF1 (with KDF1 score being 3) groups. As shown in Figure 3A, no significant difference in OS was observed between low- and high-KDF1 groups (*p* = 0.307), but a trend of worse prognosis was observed in the high-KDF1 group. The survival observation time was considered as the time from diagnosis to final follow-up. Considering that genetic background differences in different patients may affect KDF1 expression, which may interfere with our observations of the pathological significance of KDF1 expression levels, we further compared the expression levels of KDF1 in cancer cells and paired adjacent non-neoplastic lung epithelial cells; and based on whether KDF1 levels were higher in cancer cells than in adjacent non-neoplastic lung epithelial cells, patients were divided into KDF1-increasing group and KDF1-non-increasing groups. As shown in Figure 3B, we observed a significantly shorter OS in the KDF1-increasing group compared with the KDF1-non-increasing group.

### 3.5. KDF1 Promoted the Proliferation, Migration and Invasion of LUAD Cells

**Figure 4:**
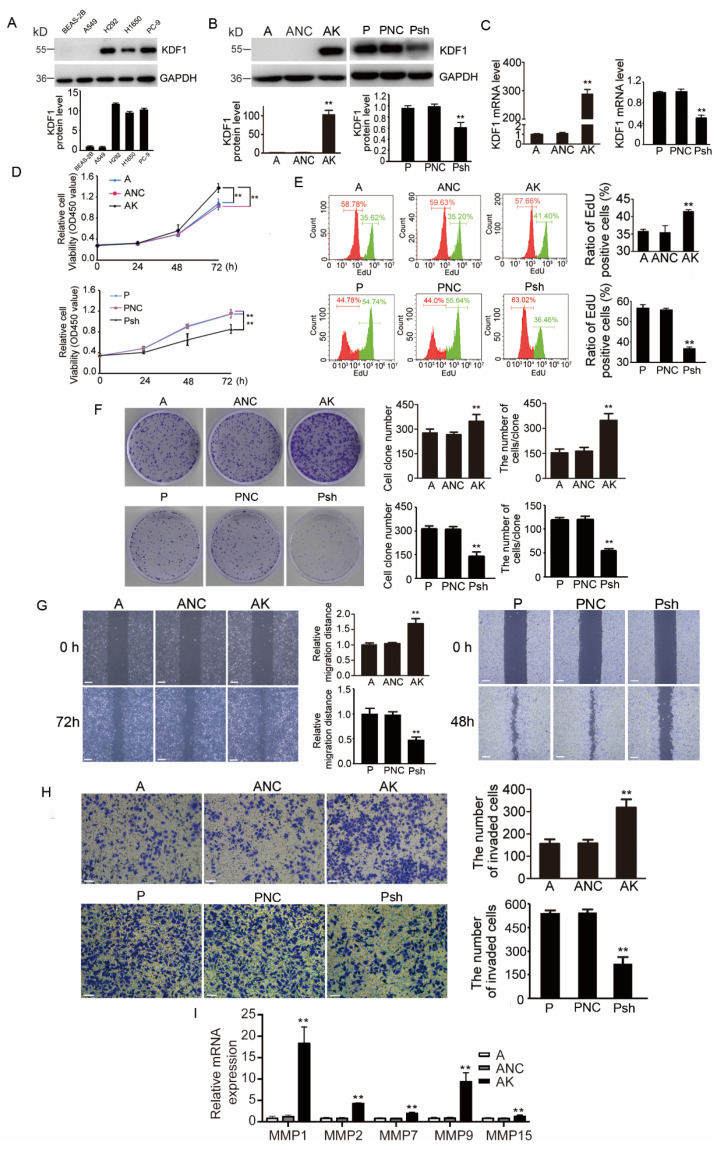
KDF1 promoted the proliferation, clone formation, migration and invasion of LUAD cells, and the expression of matrix metalloproteinases (MMPs). The KDF1 protein expression in several LUAD cell lines and normal lung epithelial cell line was detected by Western blot (**A**). Western blot (**B**) and quantitative RT-PCR (**C**) were used to confirm the expression of KDF1 in A549 or PC-9 cells. The effects of KDF1 overexpression or knockdown on the phenotype of LUAD cells were detected by CCK-8, EdU incorporation, cell clone formation, wound-healing and matrix invasion tests (**D**–**H**). The influence of KDF1 overexpression on the expression of MMPs was analyzed by quantitative RT-PCR (**I**). A: Untransfected A549 cells; ANC: A549 cells infected with the control virus; AK: A549 cells overexpressing KDF1. P: Untransfected PC-9 cells; PNC: PC-9 cells infected with the control virus; Psh: PC-9 cells with low KDF1 expression. ** *p* < 0.01 vs. A or P in part (**B**–**H**). Scale bar: 100 μm.

As shown above, KDF1 was upregulated in the cancer cells of LUAD patients and significantly correlated with tumor size and OS. To further explore the possible function of KDF1 in LUAD, in vitro experiments were performed by using the LUAD cell lines. First, we detected the expression of KDF1 protein in LUAD cell lines A549, H292, H1650 and PC-9 and normal lung epithelial cell line BEAS-2B through Western blot (Figure 4A). We found KDF1 protein was highly expressed in PC-9 cells and low in A549. Thus, these two LUAD cell lines were selected for subsequent experiments. Due to the extremely low expression of KDF1 in A549 cells (almost undetectable by Western blot), a cellular model AK that stably overexpressed KDF1 was generated by infecting the A549 cells with Lenti-KDF1. For PC-9 cells, we obtained another cell model, Psh, with stable low expression of KDF1 through Lenti-shKDF1 infection. The expression of KDF1 in the transfected cells was verified by both Western blot (Figure 4B) and qPCR (Figure 4C). According to the results of CCK-8 assay (Figure 4D), EdU incorporation analysis (Figure 4E) and colony formation assay (Figure 4F), the upregulation of KDF1 significantly promoted the proliferation rate of A549 cells, while KDF1 suppression showed a significant decrease in the proliferative ability of PC-9 cells. Furthermore, overexpression of KDF1 significantly increased the abilities of migration and invasion of A549 cells (Figure 4G,H). In addition, qPCR results showed that overexpression of KDF1 significantly increased the expression levels of MMP1, MMP2, MMP7, MMP9 and MMP15 in A549 cells (Figure 4I).

### 3.6. Re-Knockdown of KDF1 in KDF1-Overexpressing A549 Cells Reversed the Cell Phenotype

**Figure 5:**
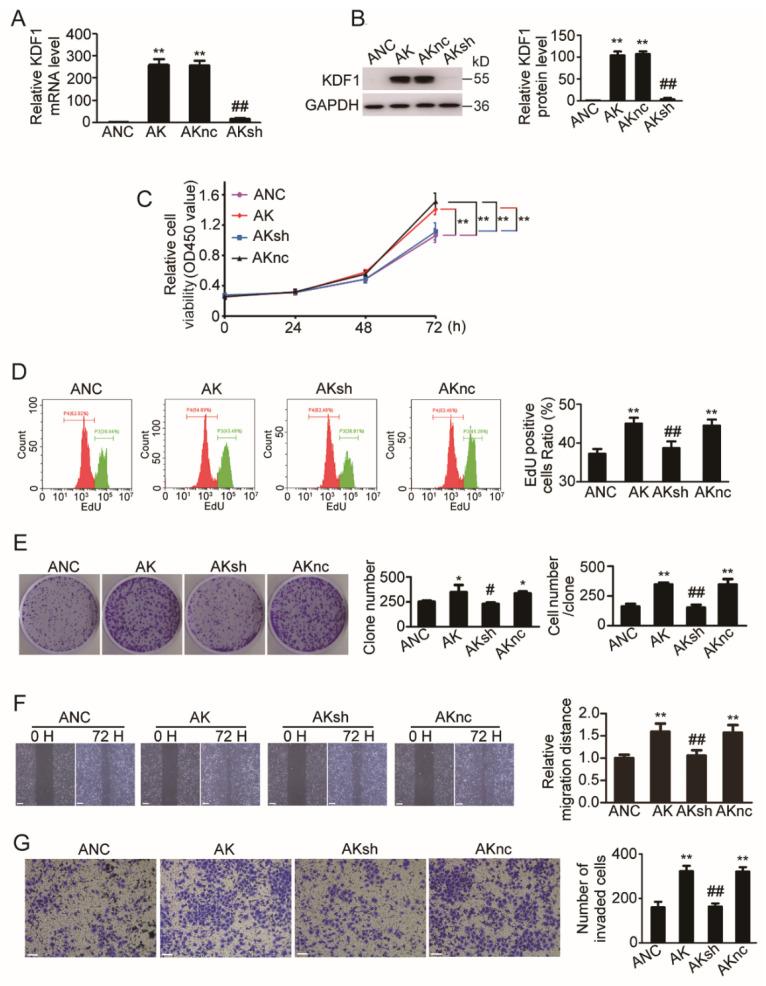
Re-knockdown of KDF1 reversed the changes in cellular phenotype caused by KDF1 overexpression in A549 cells. Re-knockdown of KDF1 was completed by infecting the KDF1-overexpressing cells with Lenti-shKDF1, a lentivirus designed to overexpress an shRNA targeting KDF1. The re-knockdown of KDF1 in cells was identified by quantitative PCR (**A**) and Western blot (**B**). CCK-8 assay (**C**), EdU incorporation assay (**D**), cell clone formation assay (**E**), wound-healing test (**F**) and Matrigel invasion assay (**G**) were used to determine the effects caused by KDF1 re-knockdown in KDF1-overexpressing A549 cells. ANC: A549 cells infected with Lenti-O-VC; AK: A549 cells overexpressing KDF1; AKsh: AK cells infected with Lenti-shKDF1; AKnc: AK cells infected with control virus Lenti-sh-VC. * *p* < 0.05 vs. ANC in part (**E**); ** *p* < 0.01 vs. ANC in other parts. # *p* < 0.05 vs. AK in part (**E**); ## *p* < 0.01 vs. AK in other parts. Scale bar: 100 μm.

To confirm that the phenotypic changes we observed in KDF1-overexpressing A549 cells were indeed caused by increased KDF1, we performed a KDF1 re-knockdown test by infecting the cells with Lenti-shKDF1. As shown in Figure 5A,B, infection of KDF1-overexpressing A549 cells by Lenti-shKDF1 significantly reduced KDF1 expression in the cells. Of note, the promoting effects induced by KDF1 overexpression on cell proliferation, clone formation, migration and invasion were almost completely reversed by re-knockdown of KDF1 (Figure 5C–G).

### 3.7. KDF1 Overexpression Significantly Upregulates the Level of AKT, p-AKT and p-STAT3 but Had No Effect on the Level of Iκκα, p38, p-p38 ERK, p-ERK and STAT3

In keratinocytes, KDF1 was reported to increase the stability of Iκκα [8]. To determine whether KDF1 also exerts its roles in A549 cells through influencing Iκκα, we examined Iκκα expression level in the KDF1-overexpressing A549 cells. No significant difference was observed in the expression level of Iκκα between the KDF1-overexpressing cells and the control group, thus excluding the possibility that, through influencing the stability of Iκκα, KDF1 exerted its role in the A549 cells.

**Figure 6:**
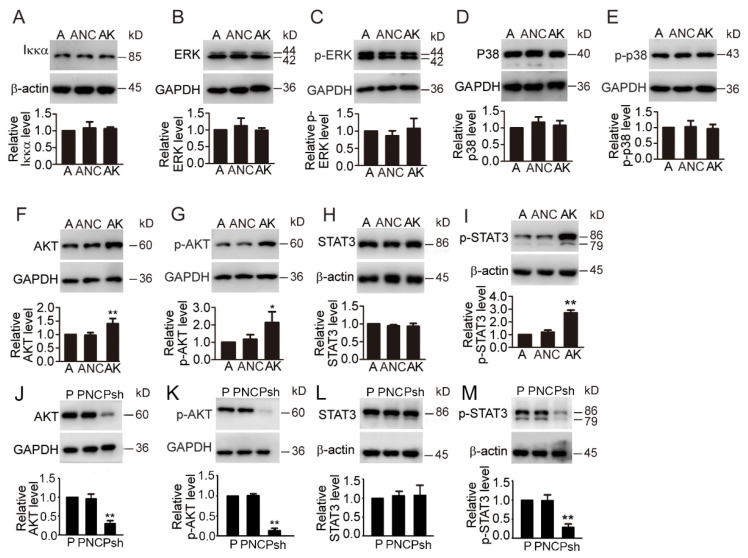
Effect of KDF1 on the cellular level of tumor-associated protein. Western blot analysis of Iκκα (**A**), ERK (**B**), phosphorylated ERK (**C**), p38 (**D**), phosphorylated p38 (**E**), AKT (**F**,**J**), phosphorylated AKT (**G**,**K**), STAT3 (**H**,**L**) and phosphorylated STAT3 (**I**,**M**) in LUAD cells. A: Uninfected A549 cells; ANC: Control-virus-infected A549 cells; AK: A549 cells overexpressing KDF1. P: Untransfected PC-9 cells; PNC: PC-9 cells infected with the control virus; Psh: PC-9 cells with low KDF1 expression. * *p* < 0.05 vs. A or P; ** *p* < 0.01 vs. A or P.

Given the importance of the ERK [9], p38 [10], AKT [11,12] and STAT3 [13,14,15] pathways in the regulation of cell proliferation, migration and invasion and the initiation and progression of lung cancer, we examined the effects of KDF1 overexpression on these pathways by detecting the expression level of AKT, p-AKT, ERK, p-ERK, p38, p-p38, STAT3 and p-STAT3 in A549 cells overexpressing KDF1. We did not observe significant changes in the cellular level of ERK, p-ERK, p38, p-P38 and STAT3 in the KDF1-overexpressing cells compared with those in the controls (Figure 6B–F). However, we observed significantly enhanced expression of AKT, p-AKT and p-STAT3 (Figure 6G–I) in A549 cells overexpressing KDF1 compared with the controls. To verify this, we also detected the effect of KDF1 depletion on the phosphorylation levels of AKT and STAT3 in PC-9 cells that naturally highly express KDF1. And the results showed KDF1 depletion significantly reduced the expression of p-AKT and p-STAT3 but had no effect on AKT and STAT3 (Figure 6J–M). Overall, we found that KDF1 is most likely to promote the proliferation, migration and invasion of LUAD cancer cells via activation of the STAT3 pathway and AKT pathway.

### 3.8. STAT3 Inhibitor Inhibited AKT Signaling Pathway and Prevented the Phenotypic Changes Induced by KDF1 Overexpression

**Figure 7:**
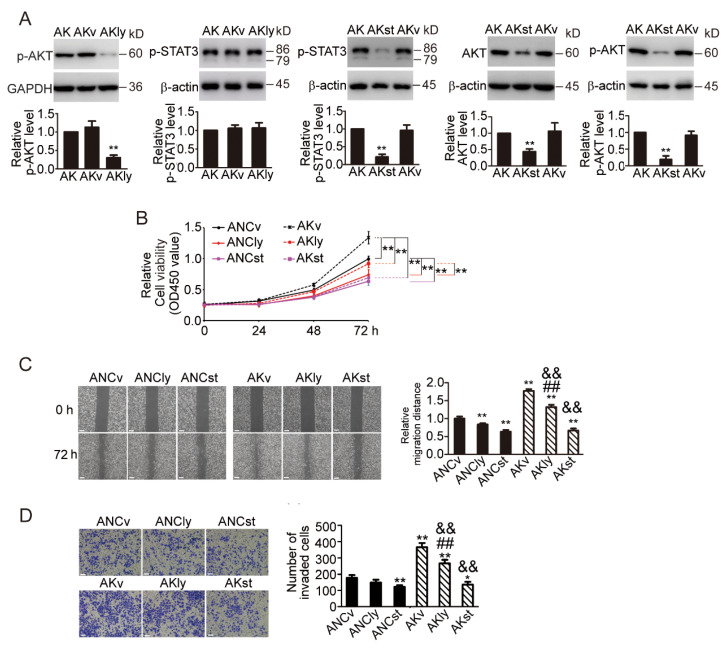
STAT3 inhibitor inhibited AKT signaling pathway and the phenotypic changes induced by KDF1 overexpression. (**A**) Stattic treatment significantly lowered the cellular level of p-STAT3, AKT, p-AKT, whereas LY294002 treatment reduced p-AKT level but did not affect the expression of p-STAT3 in A549 cells overexpressing KDF1. (**B**) Results of CCK-8 assay. (**C**) Results of wound-healing ability analysis. (**D**) Results of cell invasion ability analysis. ANC: Control-virus-infected A549 cells; AK: A549 cells overexpressing KDF1; AKly: AK cells treated with 10 μg/mL of LY294002; AKst: Stattic (5 μM)-treated AK cells; AKv: solvent (the same volume of DMSO)-treated AK cells; ANCly: ANC cells treated with 10 μg/mL of LY294002; ANCst: Stattic (5 μM)-treated ANC cells; ANCv: solvent (the same volume of DMSO)-treated ANC cells. ** *p* < 0.01 vs. AK in part (**A**); ** *p* < 0.01 in part (**B**); ** *p* < 0.01 vs. ANCv in part (**C**,**D**); * *p* < 0.05 vs. ANCv in part (**D**). &&: *p* < 0.01 vs. AKv. ##: *p* < 0.01 vs. ANCly. Scale bar: 100 μm.

To further confirm the involvement of AKT and STAT3 pathways in the function of KDF1 in LUAD cells, we treated the KDF1-overexpressing A549 cells with LY294002, an AKT pathway-specific inhibitor, and Stattic, a STAT3 pathway inhibitor. LY294002 (10 μg/mL) treatment markedly inhibited p-AKT expression but did not influence the level of p-STAT3 (Figure 7A). Treatment of the cells with Stattic (5 μM) not only significantly reduced the expression level of p-STAT3 but also downregulated the expression of AKT and p-AKT (Figure 7A), indicating that AKT is on the downstream of the STAT3 pathway.

In addition, we observed that both LY294002 and Stattic treatment significantly inhibited the promotion of KDF1 overexpression in the cellular proliferation, migration and invasion of A549 cells, and Stattic treatment appeared to have a better effect (Figure 7B–D).

### 3.9. Overexpression of KDF1 Increased Xenograft Tumor Growth

As KDF1 overexpression has been shown to increase the proliferation of LUAD cells (A549 cells) in vitro, we speculated that overexpression of KDF1 might also enhance LUAD tumor growth in vivo.

**Figure 8:**
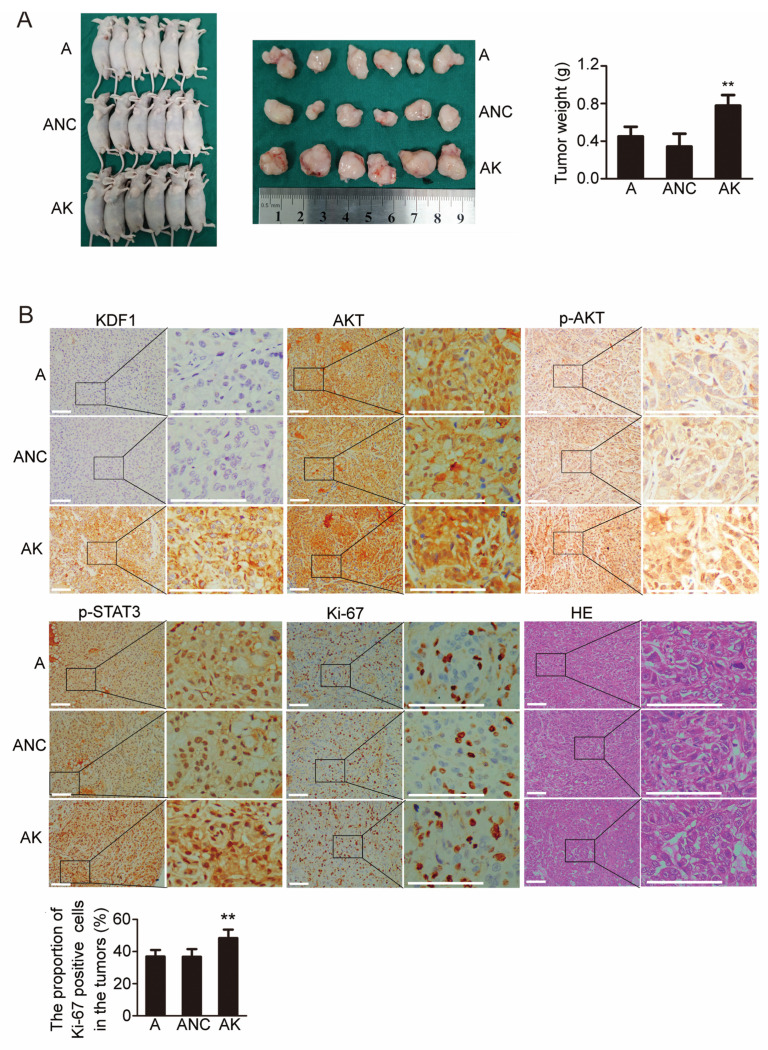
Overexpression of KDF1 increased the growth of xenograft tumors, the level of AKT, phosphorylated AKT (p-AKT) and phosphorylated STAT3 (p-STAT3) and the ratio of Ki-67-positive cells in tumors. Three groups of mice were included in the experiments (6 nude mice for each group): A549 cell group (**A**), control-virus-transfected A549 cell group (ANC) and KDF1-overexpressing A549 cell group (AK). For each mouse, a total of 1 × 10^6^ cells (untransfected A549 cells for A group, control-virus-transfected A549 cells for ANC group and A549 cells overexpressing KDF1 for AK group) were transplanted subcutaneously. Six weeks later, we euthanized the mice, harvested and weighed the tumors. Immunohistochemical staining for KDF1, AKT, p-AKT, p-STAT3 and Ki-67 and hematoxylin–eosin (HE) staining were performed on the paraffin sections of the transplanted tumors. (**A**) Results of the tumor transplantation tests. (**B**) Results of HE staining and immunohistochemistry. ** *p* < 0.01 vs A. Scale bar: 100 μm.

To test our supposition, we carried out a tumor xenograft trial. As shown in Figure 8A, tumors derived from KDF1-overexpressing A549 cells were significantly larger than tumors derived from the control cells. In the meanwhile, we observed that the level of KDF1, AKT, p-AKT and p-STAT3 and the ratio of Ki-67-positive cells were significantly increased in the tumor tissues derived from KDF1-overexpressing cells (Figure 8B). However, we did not observe morphologic differences between tumors derived from KDF1-overexpressing cells and tumors derived from control cells (Figure 8B HE staining).

### 3.10. Correlation of KDF1 and p-STAT3 Level in Human LUAD Tumor Cells

**Figure 9:**
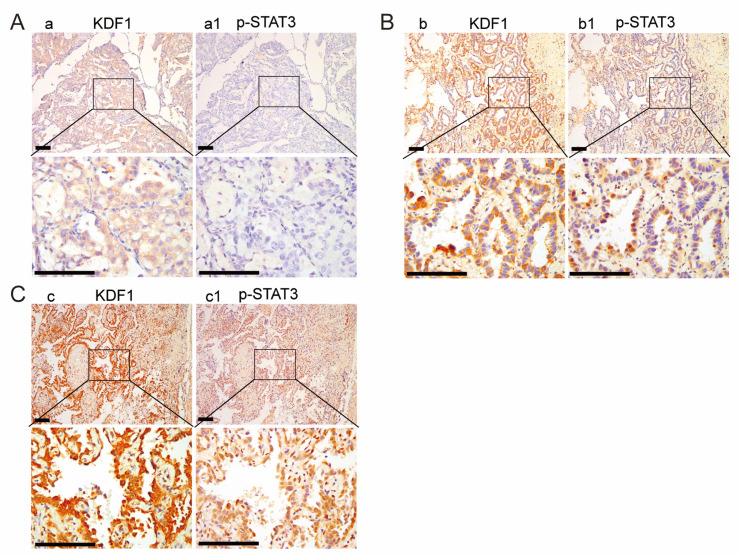
Representative expression of p-STAT3 in lung adenocarcinoma (LUAD) tumor tissues in association with that of KDF1. (**A**) Images showing that a LUAD tumor tissue sample with weak (scored as 1) level of KDF1 in the cancer cells had low (scored as 1) level of p-STAT3 in the cancer cells. (**B**) Images showing that a LUAD tumor tissue sample with medium (scored as 2) level of KDF1 in the cancer cells had medium (scored as 2) level of p-STAT3 in the cancer cells. (**C**) Images showing that a LUAD tumor tissue sample with high (scored as 3) level of KDF1 in the cancer cells had high (scored as 3) level of p-STAT3 in the cancer cells. Here, a1, b1 and c1 are serial sections from a same LUAD tumor tissue sample, respectively. Scale bar: 200 μm.

So far, evidence from our study has supported the hypothesis that the STAT3 signaling pathway may play a crucial role in the oncogenetic function of KDF1 in LUAD cell line A549. To determine whether this was also the case in the cancer cells of LUAD tumor tissues, we further examined p-STAT3 expression in the cancer cells of LUAD tumor tissue samples and associated them with the expression level of KDF1. We observed an obvious positive association between the expression level of KDF1 and that of p-STAT3: LUAD cancer cells expressing a high level of KDF1 usually express a high level of p-STAT3 and vice versa (Figure 9). To better determine the correlation between KDF1 and p-STAT3, the level of p-STAT3 and KDF1 in each tumor tissue specimen was carefully scored and then a Spearman correlation was carried out. We observed that the expression level of p-STAT3 is positively correlated with the level of KDF1 in the cancers (r = 0.458, *p* < 0.01).

## 4. Discussion

In the present study, we study the expression and the possible role of KDF1 in LUAD and the potential mechanism by which KDF1 functions. We observed that KDF1 was upregulated in LUAD tumor tissues. It was mainly expressed in the cancer cells and some non-cancer cells and distributed in the cytoplasm. Compared with the paired adjacent alveolar epithelial cells, in most cases, the cancer cells expressed a much higher level of KDF1. It was found that KDF1 expression correlated positively with the tumor size. Patients with elevated KDF1 levels in cancer cells (compared with paired alveolar epithelial cells) had shorter OS than those without elevated KDF1 levels in cancer cells. These findings suggest that KDF1 is highly associated with the pathogenesis of LUAD and may act as a protumor factor. This hypothesis was supported by our further in vitro and in vivo experiments, in which KDF1 depletion significantly inhibited the growth of LUAD cells with naturally high KDF1 expression in vitro. In contrast, ectopic expression of KDF1 significantly promoted the proliferation, clone formation, migration and invasion of LUAD cells and the growth of xenograft tumors. Additionally, we detected the cell phenotype changes in AKsh (re-knockdown of KDF1 in A549 cells overexpressing KDF1) cells, and the results showed KDF1 suppression significantly inhibited LUAD cell proliferation and migration. KDF1 may be a potential therapeutic target for LUAD.

Initially identified as a key molecule in the regulation of mouse epidermis development, KDF1 has been reported to be associated with some genetic defects of ectoderm-derived organs [16,17,18,19]. Structurally, KDF1 was predicted to be a fairly flexible protein with a few isolated secondary structures, most of which are helices, suggesting that KDF1 may act as a scaffold, adaptor or cofactor [19]. In consistence with this, in keratinocytes, KDF1 was reported to be able to bind to Iκκα and USP7, a key deubiquitination enzyme, thereby reducing the ubiquitination level of Iκκα and increasing its stability [8]. The critical role of KDF1 in controlling the equilibrium between the differentiation and proliferation of epidermal progenitor cells naturally suggests a possible role for this molecule in tumors. Indeed, as shown in this study (Figure 2), the expression of KDF1 was found to be dysregulated in most of the cancers analyzed. However, data about the involvement of KDF1 in cancers are still limited. The present finding that KDF1 may participate in the process of LUAD pathogenesis has provided us with new evidence for the involvement of this molecule in cancers.

Quite different from the role observed in epidermal progenitor cells and ccRCC cells [6], KDF1 was found to promote rather than inhibit the proliferation of epithelial ovarian cancer cells [7] and LUAD cells in a study of Zhu et al. and our present study, respectively. This phenomenon may be explained by the molecular function of KDF1 as a scaffold, adaptor or cofactor, since different cellular contexts may mean different molecular partners present that interact with KDF1.

The previous findings that KDF1 could increase the stability of Iκκα are attractive for us to explore the mechanism of KDF1 in LUAD. To determine whether Iκκα was involved in the function of KDF1 in LUAD, we investigated the protein level of Iκκα in KDF1-overexpressing cells. But we did not observe a significant difference in the level of Iκκα in KDF1-overexpressing and non-overexpression LUAD cells, thus excluding the possibility that KDF1 exerts its role in LUAD cells through influencing the level of Iκκα. This finding also emphasized the cell context-dependent nature of KDF1 action. In consistence with this, a previous study showed that only upon differentiation (but not in other conditions) did KDF1 display its role in maintaining the stability of Iκκα in keratinocytes [8].

According to a study of Pezzuto A et al. [20], KDF1 may participate in the process of inducing the expression of other tumor signaling pathway proteins. Given the critical roles of p38 [10], ERK [9], AKT [11,12] and STAT3 [13,14,15] pathways in lung cancer’s initiation and progression, we further examined the influence of KDF1 overexpression in these pathways. And we observed KDF1 expression significantly affected the expression levels of p-AKT and p-STAT3 in LUAD cells but did not observe significant differences in the levels of p38, p-p38, ERK, p-ERK and STAT3 in KDF1-overexpressing and non-overexpressing cells. Increased expression of AKT, p-AKT and p-STAT3 was also present in the xenograft tumors derived from KDF1-overexpressing cells. Moreover, we observed that inhibition of the AKT or STAT3 pathway significantly reduced the phenotypic changes induced by KDF1 overexpression in A549 cells, and inhibition of the STAT3 pathway appeared to have a better effect. In addition, we observed that treatment of the cells with STAT3-specific inhibitors not only reduced the activation level of the STAT3 pathway (represented by decreased p-STAT3 level) but also significantly reduced the expression and activation level of AKT. In contrast, treatment of the cells with a specific inhibitor of the AKT pathway only decreased p-AKT level and did not affect the activation of the STAT3 pathway. These results indicate that, in KDF1-overexpressing LUAD cells, the STAT3 pathway is on the upstream of the AKT pathway and, by activating the STAT3 pathway and later AKT and other STAT3 downstream pathways, the expression levels of KDF1 in the cancer cells correlated positively to the levels of p-STAT3 in the cells. Meanwhile, STAT3 protein increased with the stages of tumor patients [21]. These results further supported our idea that KDF1 exerted its protumor role in LUAD through activating the STAT3 pathway.

Initially identified as an IL-6-induced acute phase response protein, STAT3 has been extensively investigated and is reported to participate in many physiological and pathological processes [22,23,24,25,26,27]. It is known that, in oncology, signals mediated by STAT3 contribute to the onset and progression of malignancies [27]. STAT3 was frequently found to be constitutively activated in many cancers and was believed to contribute to the progression of cancers either through its direct roles on cancer cells, such as promoting survival, preventing apoptosis and increasing proliferation, migration and invasion of cancer cells, or through its indirect roles in the regulation of antitumor immune response [28]. Constitutive activation of STAT3 was observed in more than 50% of non-small-cell lung carcinoma (NSCLC) patients [27] and correlated with tumor lymph node metastasis, drug resistance, advanced clinical stage and low level of tumor differentiation [27,29,30,31]. In addition, enhanced activation of STAT3 was reported to promote cancer cell stemness and the immune evasion of lung cancers [27,32,33,34,35]. Due to the key roles of the STAT3 pathway, it is considered as a promising therapeutic target for lung cancer treatments. Combined with relevant research, we propose that inhibition of KDF1 or STAT3 may be a novel therapeutic strategy for LUAD with high expression of KDF1.

It should be pointed out that in the present study, we did not answer how KDF1 promoted the activation of the STAT3 pathway. Further work is needed to answer this question. In addition, the number of patients involved in this study is small, and further in-depth studies based on a larger range of populations are still needed to dissect the exact clinical significance of KDF1 in LUAD.

## 5. Conclusions

In summary, this study demonstrated the expression and possible role of KDF1 in LUAD as well as the potential mechanism. KDF1 was upregulated in LUAD tissues and might contribute to the progression of LUAD by enhancing the proliferation, migration and invasion of LUAD cancer cells via activation of the STAT3 pathway and AKT pathway. Targeting KDF1 might be a potential strategy for the treatment of the disease.

## Figures and Tables

**Table 1:** Comparison of KDF1 level in LUAD cancer cells of different groups.

Variable	KDF1 Level in Tumor Cells (Immunostaining Score)			*p*
	1 Number (%)	2 Number (%)	3 Number (%)	
Age (years)				
≤median	17 (21.0)	31 (38.3)	33 (40.7)	0.724
>median	15 (19.0)	36 (45.6)	28 (35.4)	
Gender				
Male	9 (16.1)	25 (44.6)	22 (39.3)	0.562
Female	23 (22.1)	42 (40.4)	39 (37.5)	
Tumor size				
≤median	22 (27.8)	34 (43.0)	23 (29.1)	0.010
>median	10 (12.8)	33 (42.3)	35 (44.9)	
T (primary tumor extent)				
T0 or T1	25 (24.0)	41 (39.4)	38 (36.5)	0.227
T2–4	7 (12.5)	26 (46.4)	23 (41.1)	
With lymph node or distant metastasis				
Yes	4 (13.8)	10 (31.0)	15 (55.2)	0.104
No	28 (21.4)	57 (43.5)	46 (35.1)	
TNM stage				
Carcinoma in situ/I	27 (21.4)	56 (44.4)	43 (34.1)	0.065
II–IV	5 (14.7)	11 (32.4)	18 (52.9)	

## Data Availability

The data analyzed during the present study are available from the corresponding author on reasonable request.

## Abbreviations

LUAD: lung adenocarcinoma; OS, overall survival; KDF1, keratinocyte differentiation factor 1; MOI, multiplicity of infection factor 1; qPCR, quantitative real-time polymerase chain reaction; SDS-PAGE, sodium lauryl sulfate polyacrylamide gel electrophoresis; PVDF, polyvinylidene fluoride; p-ERK, phosphorylated ERK; p-p38, phosphorylated p38; CCK-8, Cell Counting Kit-8; p-AKT, phosphorylated AKT; p-STAT3, phosphorylated STAT3; CESC, cervical squamous cell carcinoma and endocervical adenocarcinoma; BRCA, breast-invasive carcinoma; ESCA, esophageal carcinoma; STES, stomach and esophageal carcinoma; STAD, stomach adenocarcinoma; PRAD, prostate adenocarcinoma; UCEC, uterine corpus endometrial carcinoma; HNSC, head and neck squamous cell carcinoma; LUSC, lung squamous cell carcinoma; BLCA, bladder urothelial carcinoma; CHOL, cholangiocarcinoma; GBM, glioblastoma multiforme; GBMLGG, glioma; LGG, brain lower-grade glioma; COAD, colon adenocarcinoma; COADREAD, colon adenocarcinoma/rectum adenocarcinoma esophageal carcinoma; KIRP, kidney renal papillary cell carcinoma; KIPAN, pan-kidney cohort (KICH + KIRC + KIRP); KIRC, kidney renal clear cell carcinoma; PCPG, pheochromocytoma and paraganglioma; KICH, kidney chromophobe; HE, hematoxylin–eosin; NSCLC, non-small-cell lung carcinoma.
